# Changes in Trends of Shoulder and Knee Arthroscopy Because of the COVID-19 Pandemic

**DOI:** 10.5435/JAAOSGlobal-D-25-00178

**Published:** 2026-02-11

**Authors:** Rashmi Pathak, Andrea L. Aagesen, Georges Bounajem, Lichen Du, Gregory D. Ayers, Michael Khazzam, Folefac D. Atem, Nitin B. Jain

**Affiliations:** From the Departments of Physical Medicine and Rehabilitation (Pathak, Dr. Aagesen, and Dr. Jain) and the Department of Orthopaedics (Dr. Jain), University of Michigan, Ann Arbor, MI; the Department of Physical Medicine and Rehabilitation, University of Texas Southwestern, Dallas, TX (Dr. Du); the Department of Biostatistics, Vanderbilt University School of Medicine, Nashville, TN (Dr. Ayers); the Departments of Orthopaedics Surgery, University of Texas Southwestern, Dallas, TX (Dr. Bounajem, and Dr. Khazzam); and the Department of Biostatistics and Data Science, University of Texas Health Science Center-Houston, Houston, TX (Dr. Atem).

## Abstract

**Purpose:**

The purpose of this study was to analyze national trends in knee and shoulder arthroscopy to identify seasonal and annual variability using the Healthcare Cost and Utilization Project-Nationwide Ambulatory Surgery Sample (HCUP-NASS) dataset.

**Methods:**

Estimated national rates of ambulatory knee and shoulder arthroscopy were analyzed using HCUP-NASS data from 2016 to 2021. Time trend plots were generated to identify potential seasonal trends in these procedures.

**Results:**

There was a decreasing trend in the number of procedures (shoulder arthroscopy, rotator cuff repair [RCR], knee arthroscopy, meniscal repair [MR]/meniscectomy, and anterior cruciate ligament reconstruction [ACLR]) done from 2016 to 2021. There were an estimated 264,987 (95% confidence interval [CI] = 251,205 to 276,770) patients who underwent shoulder arthroscopy and 381,125 (95% CI = 362,555 to 399,696) patients who underwent knee arthroscopy in 2021, as compared with 345,892 (95% CI = 326,224 to 365,559) patients who underwent shoulder arthroscopy and 521,912 (95% CI = 496,905 to 546,919) patients who underwent knee arthroscopy in 2016. Male patients showed markedly higher rates of shoulder arthroscopy, RCR, knee arthroscopy, MR, and ACLR procedures as compared with female patients, whereas female patients showed markedly higher rates of knee arthroscopy done without MR and ACLR. Shoulder and knee arthroscopy rates peaked in the 55 to 75-year-old age group. In addition, both procedures were most frequently done in December and had the lowest utilization in the month of July.

**Conclusion:**

There was a nonlinear decrease in the estimates of shoulder arthroscopy, RCR, MR/meniscectomy, knee arthroscopy, and anterior cruciate ligament reconstruction procedures from 2016 to 2021, with peaks in 2016 and 2019, and a low point in 2020. There are notable variations in rates of knee and shoulder arthroscopy procedures by age and sex.

**Clinical relevance:**

Recent data on commonly done ambulatory orthopaedic arthroscopies are important for policy makers and for understanding utilization trends.

Knee and shoulder arthroscopies are some of the most frequently done orthopaedic surgeries and are leading reasons for patients to pursue ambulatory surgery.^1,2^ According to the Centers for Disease Control and Prevention National Survey of Ambulatory Surgery, an estimated 272,148 patients underwent rotator cuff repair (RCR), and another 257,541 underwent shoulder arthroscopy (excluding RCR) in the United States in 2006.^2^ Although studies have reported recent diverging trends in knee and shoulder arthroscopy rates at the state level, contemporary national estimates from reliable data sources are lacking. Analyzing this data would help to inform policymakers and funding agencies to leverage surgery utilization rates for the healthcare system including healthcare planning and cost management.^3-6^

The purpose of this study was to analyze national and seasonal trends in ambulatory knee and shoulder arthroscopy using the most recent available Healthcare Cost and Utilization Project-Nationwide Ambulatory Surgery Sample (HCUP-NASS) dataset from 2016 to 2021. The HCUP database is designed to represent a larger universe, so each facility's sampling encounter volume is weighted within the stratum volume to achieve national and regional estimates. We hypothesized that the number and rate of ambulatory (knee and shoulder arthroscopy) surgical procedures have been decreasing over the past several years, with a precipitous decline in 2020 because of COVID-19.

## Methods

In this cross-sectional study, we analyzed a stratified cluster sample of major ambulatory surgery encounters in the United States using data from the Nationwide Ambulatory Surgery Sample (NASS), 2016 to 2021. Thirty-five HCUP partner organizations contributed to NASS, making it the largest all-payer ambulatory surgery database in the United States. It contains approximately 760,000 records from approximately 3,000 hospital-owned facilities across 34 states and the District of Columbia, covering 83% of the total US resident population.^7^ The NASS represents an estimated 68% stratified sample of hospital-owned facilities and an estimated 76% stratified sample of ambulatory surgery encounters nationwide.^7^ Stratification is based on characteristics such as geographic region, hospital bed size, urban-rural location, hospital teaching status, and hospital ownership. Within each stratum, samples of ambulatory surgery encounters are systematically and randomly selected based on facility characteristics. Encounters are selected at fixed intervals from a randomized starting point,^8^ ensuring an unbiased selection process that accurately reflects the characteristics of each hospital stratum. Encounter weights were created by integrating the NASS universe of hospitals and encounters with the NASS sample hospitals and encounters to estimate national trends. Basic encounter and patient characteristic information was contained in the “Encounter” file, whereas stratified information of hospitals could be found in the “Hospital” file, used in conjunction with sampling weights to generate estimates. Analysis for this study was conducted using NASS “Encounter” and the “Hospital” files from the year 2016 to 2021 in patients age 18 years or older. The NASS dataset contains Current Procedural Terminology (CPT) codes developed by the American Medical Association.^9^ Inclusion criteria included patient encounters with primary or secondary CPT codes listed for knee arthroscopy and shoulder arthroscopy, in patients age 18 years or older^10^ (Table 1). Exclusion criteria include encounters with CPT codes for arthroscopic fracture repairs involving the intercondylar spine, tuberosity fracture, tibial plateau, unicondylar or bicondylar, and unlisted arthroscopic procedures and patients younger than 18 years of age.

**Table 1:** Current Procedural Terminology Codes Used for Shoulder and Knee Arthroscopy Procedures^9^

Procedures	Category	Subcategory	CPT codes
Shoulder arthroscopy	Rotator cuff repair (RCR)		29827
	Shoulder (except RCR)	Superior labrum anterior and posterior (SLAP) lesion repair, capsulorrhaphy, subacromial decompression, claviculectomy, débridement, and other shoulder arthroscopies	29805, 29806, 29807, 29819, 29820, 29821, 29822, 29823, 29824, 29825, 29826, 29828
Knee arthroscopy	Meniscal repair (MR)	Meniscectomy or meniscal repair	29880, 29881, 29882, 29883
	Anterior cruciate ligament (ACL)	Anterior cruciate ligament/posterior cruciate ligament repair or augmentation or reconstruction	29888, 29889
	Knee (except MR and ACL)	Abrasion arthroplasty/microfracture, chondroplasty, synovectomy, other knee arthroscopies	29866, 29867, 29868, 29870, 29871, 29873, 29874, 29875, 29876, 29877, 29879, 29884, 29885, 29886, 29887

### Cohort Data

Cohort data were obtained from the American Factfinder website for 2016 to 2021.^11^ The cohort was stratified by age and sex to calculate procedure rates per 100,000 persons.

### Statistical Analysis

The “Encounter” dataset was merged with the “Hospital” dataset using a common variable. Age was categorized into four groups: 18 to 34 years, 35 to 54 years, 55 to 74 years, and 75 years and older. The estimated total number of knee arthroscopy, shoulder arthroscopy, arthroscopic RCR, meniscal repair (MR)/meniscectomy, and anterior cruciate ligament (ACL) reconstruction procedures and the procedure rates (per 100,000 persons) from 2016 to 2021, were calculated. Weighted variance and 95% confidence intervals were determined using statistical programming from HCUP.^12^ SAS 9.4 software^13^ was used for NASS data filtering, cleaning, and national estimation, whereas R 4.0.4^14^ was used for statistical analyses and data visualization. The SURVEYMEANS procedure was used to obtain estimates, rates, and 95% confidence intervals across the year 2016 to 2021. SURVEYMEANS is a linear approximation method used to estimate sampling errors based on complex sample design with stratification (by sex and age), clustering (by hospital), and using weights.^15^ In addition, the nonparametric Mann-Kendall test was used to statistically assess trends over time. A *p*-value of <0.05 was considered statistically significant.

## Results

### Annual Trends in Arthroscopic Knee Surgeries

In 2016, an estimated 415,905 (95% CI = 396,429 to 435,382) patients underwent MR, 65,977 (95% CI = 61,244 to 70,711) patients underwent ACLR, and another 75,565 (95% CI = 70,980 to 80,150) patients underwent other knee arthroscopy (except MR and ACLR; Table 1). The number of all arthroscopic knee procedures declined from 521,912 (95% CI = 496,905 to 546,919) in 2016 to 437,244 (95% CI = 416,621 to 457,867) in 2018, where MR consisted of 349,554 (95% CI = 332,937 to 366,171) of that total. The lowest number of surgeries occurred in the third quarter of 2018. After this, there was a spike in 2019 in the estimate of patients undergoing all knee arthroscopy 495,342 (95% CI = 472,238 to 518,446) and the subgroup including MR at 335,883 (95% CI = 320,449 to 351,317), although both remained lower than the 2016 peak. In the first and second quarters of 2020, there was a sharp drop in the estimated number of all arthroscopic knee surgeries at 364,040 (95% CI = 347,161 to 380,921), with MR comprising 258,152 (95% CI = 246,400 to 269,903) and knee arthroscopy (except MR and ACLR) totaling 70,022 (95% CI = 65,875 to 74,170).

In 2021, there was a slight increase in all categories: 267,313 (95% CI = 254,418 to 280,210) patients underwent MR, 41,694 (95% CI = 38,642 to 44,746) patients underwent ACLR, and another 72,118 (95% CI = 67,778 to 76,458) patients underwent knee arthroscopy (except MR and ACLR; Table 1). Patients undergoing ACLR demonstrated less variability with a gradual decline from the 2016 peak of 65,977 (95% CI = 61,244 to 70,711) to 2018 at 57,704 (95% CI = 53,939 to 61,470), then a 1-year increase in 2019 to 62,077 (95% CI = 57,822 to 66,333). The estimated number of patient encounters with ACLR sharply declined in the first and second quarters of 2020 to 35,867 (95% CI = 33,324 to 38,410) and followed by a gradual increase to 41,694 (95% CI = 38,642 to 44,746) at the study conclusion in 2021.

### Annual Trends in Arthroscopic Shoulder Surgeries

In 2016, an estimated 169,275 (95% CI = 159,347 to 179,203) patients underwent RCR, and another 176,617 (95% CI = 165,924 to 187,309) patients underwent other shoulder arthroscopy (except RCR; Tables 2 and 3). The number of shoulder arthroscopies declined from 2016 through 2018, where there was an estimated 163,293 (95% CI = 154,328 to 172,257) RCR and another estimated 156,407 (95% CI = 147,704 to 165,109) underwent other shoulder arthroscopy (except RCR). In 2019, there was an increase in shoulder arthroscopy (except RCR) to 186,293 (95% CI = 176,061 to 196,525) patients, whereas the estimated number of patients who underwent a RCR continued to decline to 138,106 (95% CI = 130,332 to 145,879) in 2019 (Tables 2 and 3). In the first two quarters of 2020, there was a sharp decline in the estimated total shoulder arthroscopies 257,956 (95% CI = 244,854 to 271,060) which was a decrease from 324,399 (95% CI = 307,963 to 340,835) in the preceding year. The subset of patient encounters with RCR declined less precipitously, reaching 120,615 (95% CI = 113,857 to 127,373) in 2020. In 2021, there was a gradual rise in shoulder arthroscopies with an estimated 123,839 (95% CI = 116,848 to 130,831) patients who underwent RCR, whereas another 141,148 (95% CI = 132,946 to 149,350) patients underwent other shoulder arthroscopy (except RCR; Tables 2 and 3).

**Table 2:** Number of Procedures for Shoulder and Knee Arthroscopy by Sex

Procedure	Sex	2016		2017		2018		2019		2020		2021	
		N^a^	95% Confidence interval	N^a^	95% Confidence interval	N^a^	95% Confidence interval	N^a^	95% Confidence interval	N^a^	95% Confidence interval	N^a^	95% Confidence interval
Shoulder arthroscopy	Female	149162	140174-158150	143843	136408-151278	138587	131463-145710	140207	133220-147194	115652	109909-121396	118628	112583-124673
	Male	196607	185711-207502	191124	180880-201368	181106	171531-190681	184192	174587-193797	142304	134945-149664	146359	138622-154097
	Total	345892	326224-365559	334980	317441-352518	319699	303148-336250	324399	307963-340835	257956	244854-271060	264987	251205-276770
Shoulder (except RCR^b^)	Female	74184	69406-78961	69794	65917-73670	66257	62532-69982	79416	75084-83748	62139	58710-65568	63878	60260-67496
	Male	102371	96291-108451	97004	91451-102556	90146	85051-95241	106877	100842- 112913	75202	70916-79489	77270	72686-81854
	Total	176617	165924-187309	166803	157493-176113	156407	147704-165109	186293	176061-196525	137341	129626-145057	141148	132946-149350
Rotator cuff repair	Female	74978	70324-79632	74050	69980-78119	72330	68369-76290	60791	57375-64207	53513	50555-56471	54750	51685-57815
	Male	94236	88837-99634	94120	88836-99403	90960	85856-96064	77315	72878-81751	67102	63302-70902	69089	65163-73016
	Total	169275	159347-179203	168177	158923-177431	163293	154328-172257	138106	130332-145879	120615	113857-127373	123839	116848-130831
Knee arthroscopy	Female	258098	245857-270339	236889	226048-247730	217542	207538-227545	244452	233381-255524	180400	172314-188487	190212	181193-199232
	Male	263691	250642-276739	241721	229917-253524	219684	208833-230535	250890	238703-263077	183640	174847-192434	190913	181362-200464
	Total	521912	496905-546919	478626	456223-501028	437244	416621-457867	495342	472238-518446	364040	347161-380921	381125	362555-399696
Knee (except MR^c^ and ACL^d^)	Female	44646	41969-47324	41206	38873-43538	36633	34617-38649	55981	52997-58965	39934	37629-42239	41566	39134-43998
	Male	30901	28907-32896	28976	27240-30712	25303	23888-26718	41401	39053-43748	30088	28246-31931	30552	28644-32460
	Total	75565	70980-80150	70184	66207-74160	61944	58600-65288	97382	92128-102636	70022	65875-74170	72118	67778-76458
MR/meniscectomy	Female	199592	190387-208797	182727	174347-191107	168794	160969-176619	159477	152366-166587	124014	118581-129447	128911	122876-134947
	Male	216218	205695-226741	197462	187883-207040	180749	171745-189753	176406	167935-184878	134138	127819-140456	138402	131542-145263
	Total	415905	396429-435382	380203	362463-397943	349554	332937-366171	335883	320449-351317	258152	246400-269903	267313	254418-280210
Anterior cruciate ligament reconstruction	Female	26822	24683-28962	25883	24156-27610	24279	22682-25676	28994	26989-30999	16453	15297-17608	19735	18290-21181
	Male	39145	36432-41858	37115	34581-39648	33422	31170-35673	33083	30768-35398	19414	18027-20802	21959	20352-23565
	Total	65977	61244-70711	62999	58820-67178	57704	53939-61470	62077	57822-66333	35867	33324-38410	41694	38642-44746

ACL = anterior cruciate ligament, MR = meniscal repair, RCR = rotator cuff repair

a Estimates or sum of procedure.

b Rotator cuff repair.

c Meniscal repairs/meniscectomy.

d Anterior cruciate ligament reconstruction.

**Table 3:** Number of Procedures for Shoulder and Knee Arthroscopy by Age Group

Procedure	Age Group	2016		2017		2018		2019		2020		2021	
		N^a^	95% Confidence Interval	N^a^	95% Confidence Interval	N^a^	95% Confidence Interval	N^a^	95% Confidence Interval	N^a^	95% Confidence Interval	N^a^	95% Confidence Interval
Shoulder arthroscopy	18-35	34750	32292-37209	32251	30137-34365	29285	27404-31165	30997	28938-33056	20394	19084-21704	21372	20005-22739
	35-55	128035	120828-135242	120433	113949-126916	110654	104672-116637	105366	99872-110861	85873	81366-90380	87121	82428-91814
	55-75	167530	157974-177086	167547	158800-176293	164600	156086-173114	164467	156260-172674	140106	133127-147086	144231	136867-151595
	Older than 75	15576	14362-16791	14749	13827-15672	15160	14262-16058	14919	14124-15713	11583	10929-12238	12263	11559-12967
	Total	345892	326224-365559	334980	317441-352518	319699	303148-336250	315749	299763-331735	257956	244506-271408	264987	250859-279115
Rotator cuff repair	18-35	3096	2559-3633	2965	2601-3329	2450	2249-2652	1906	1763.4-2048.6	1740	1612-1866	1749	1612-1886
	35-55	53819	50688-56951	51378	48480-54275	47707	44993-50421	39255	37023-41487	35201	33202-37200	35425	33423-37426
	55-75	102277	96305-108249	104122	98364-109881	103129	97375-108884	88473	83395-93550	76983	72650-81315	79692	75150-84234
	Older than 75	10083	9323-10842	9711	9061-10362	10006	9346-10666	8336	7804.3-8867	6693	6247-7139	6973	6522-7425
	Total	169275	159347-179203	168177	158923-177431	163293	154328-172257	137970	130202-145737	120617	113711-127520	123839	116707-130971
Knee arthroscopy	18-35	98389	92546-104233	92193	86724-97661	84271	79243-89298	88581	83153-94010	69768	65727-73810	79021	74377-83665
	35-55	211327	201100-221553	190439	181294-199585	171190	162841-179539	175265	166790-183741	145114	138286-151941	149506	142101-156910
	55-75	194819	185341-204297	179785	171392-188179	166375	158552-174197	170662	163045-178280	137855	131603-144107	140787	134059-147515
	Older than 75	17376	16304-18449	16209	15272-17145	15408	14585-16232	15707	14948-16466	11304	10721-11887	11812	11181-12443
	Total	521912	496905-546919	478626	456223-501028	437244	416621-457867	450215	429126-47306	364041	346337-381745	381126	361718-400533
Meniscal repair/meniscectomy	18-35	57577	54140-61014	54295	51079-57512	49767	46739-52795	40154	37595-42714	31572	29760-33383	36033	33887-38178
	35-55	166889	158895-174884	149416	142260-156573	135643	128957-142329	121569	115755-127382	101752	97083-106421	103694	98626-108761
	55-75	175760	167443-184077	161838	154161-169516	150241	143026-157456	142384	135913-148856	115221	109907-120535	117616	111892-123341
	Older than 75	15678	14812-16544	14653	13806-15501	13903	13137-14668	13186	12516-13856	9607	9088-10126	9971	9411-10530
	Total	415905	396429-435382	380203	362463-397943	349554	332937-366171	317293	302663-331924	258152	2455838-270465	267314	253816-280810
Anterior cruciate ligament reconstruction	18-35	38859	36271-41447	37723	35195-40250	34530	32193-36867	27529	25522-29537	20963	19501-22425	24969	23199-26738
	35-55	23293	21672-24914	22085	20560-23610	20150	18794-21506	16315	15092-17539	13032	12102-13962	14694	13595-15793
	55-75	3623	2507-4740	3129	2765-3493	2988	2726-3251	2192	1952-2433	1861	1667-2055	2020	1781-2259
	Older than 75	202	538	63	9-116	36	20-70	59.8	42-78	11	4-18	12	3-21
	Total	65977	61244-70711	62999	58820-67178	57704	53939-61470	46095.8	42748-49446	35867	33274-38460	41695	38578-44811

a Estimates or sum of procedure.

### Arthroscopic Procedure Rates

There was a double U-shaped trend in the rate of shoulder arthroscopy and knee arthroscopy, including MR/meniscectomy. There was an initial decline starting in 2016, reaching an inflection point in 2018, followed by a subsequent rise in 2019. This was then followed by a sharp decline in 2020, and a recovery trend emerged in 2021. Conversely, the ACLR and RCR demonstrated a nonlinear decrease in the rate of procedures from 2016 through 2021, with a sharp drop in 2020 followed by a slight rise in 2021. Overall, the rates of shoulder and knee arthroscopy markedly decreased from 139.64 to 103.71 (per 100,000 persons) and 210.7 to 149.17 (per 100,000 persons), respectively, from 2016 to 2021 (Tables 4 and 5). Furthermore, MR comprised most of the knee arthroscopy procedures across years, with 79.69% in 2016 and 70.14% in 2021. Rotator cuff repair comprised most of the shoulder arthroscopy procedures across years, with 48.93% in 2016 and 46.73% in 2021.

**Table 4:** Rate of Procedures for Shoulder and Knee Arthroscopy by Sex

Procedure	Sex	2016		2017		2018		2019		2020		2021	
		Procedure Rate	95% Confidence Interval	Procedure Rate	95% Confidence Interval	Procedure Rate	95% Confidence Interval	Procedure Rate	95% Confidence Interval	Procedure Rate	95% Confidence Interval	Procedure Rate	95% Confidence Interval
Shoulder arthroscopy	Female	117.21	110.15-124.27	111.98	106.19-117.77	107.11	101.6-112.61	107.58	102.22-112.94	89.02	84.60-93.44	91.05	86.41-95.69
	Male	163.23	154.18-172.28	157.06	148.64-165.48	147.65	139.84-155.45	149.01	141.24-156.78	115.35	109.38-121.32	116.88	110.70-123.06
	Total	139.64	131.7-147.58	133.92	126.91-140.93	126.84	120.27-133.41	127.75	121.27-134.22	101.84	96.67-107.02	103.71	98.32-108.32
Shoulder (except RCR^a^)	Female	58.29	54.54-62.05	54.33	51.32-57.35	51.21	48.33-54.09	60.93	57.61-64.26	47.83	45.19-50.47	49.03	46.25-51.80
	Male	84.99	79.94-90.04	79.72	75.15-84.28	73.49	69.34-77.65	86.46	81.58-91.35	60.96	57.48-64.43	61.70	58.04-65.37
	Total	71.3	66.98-75.62	66.68	62.96-70.41	62.05	58.6-65.51	73.36	69.33-77.39	54.22	51.18-57.27	55.24	52.03-58.45
Rotator cuff repair	Female	58.92	55.26-62.57	57.65	54.48-60.82	55.9	52.84-58.96	46.64	44.02-49.26	41.19	38.91-43.46	42.02	39.67-44.37
	Male	78.24	73.76-82.72	77.35	73-81.69	74.16	69.99-78.32	62.55	58.96-66.14	54.39	51.31-57.47	55.17	52.04-58.31
	Total	68.34	64.33-72.34	67.23	63.53-70.93	64.79	61.23-68.34	54.39	51.32-57.45	47.62	44.95-50.29	48.47	45.73-51.20
Knee arthroscopy	Female	202.81	193.19-212.43	184.42	175.98-192.86	168.13	160.4-175.86	187.56	179.07-196.06	138.86	132.64-145.08	146	139.08-152.93
	Male	218.93	208.09-229.76	198.64	188.94-208.34	179.1	170.25-187.95	202.97	193.11-212.83	148.86	141.73-155.99	152.46	144.84-160.09
	Total	210.7	200.6-220.79	191.34	182.39-200.3	173.48	165.29-181.66	195.06	185.97-204.16	143.73	137.07-150.39	149.17	141.90-156.44
Knee (except MR^b^ and ACL^c^)	Female	35.08	32.98-37.19	32.08	30.26-33.89	28.31	26.75-29.87	42.95	40.66-45.24	30.73	28.96-32.51	31.90	30.03-33.77
	Male	25.66	24-27.31	23.81	22.39-25.24	20.63	19.48-21.78	33.49	31.59-35.39	24.39	22.89-25.88	24.39	22.87-25.92
	Total	30.51	28.65-32.36	28.06	26.47-29.65	24.58	23.25-25.9	38.35	36.28-40.42	27.64	26-29.28	28.22	26.52-29.92
MR/meniscectomy	Female	156.84	149.6-164.07	142.25	135.73-148.77	130.45	124.41-136.5	122.36	116.91-127.82	95.46	91.27-99.64	98.95	94.31-103.58
	Male	179.51	170.78-188.25	162.27	154.4-170.14	147.36	140.02-154.7	142.71	135.86-149.57	108.73	103.61-113.85	110.53	105.05-116.01
	Total	167.9	160.04-175.76	152	144.9-159.09	138.69	132.09-145.28	132.27	126.19-138.35	101.92	97.28-106.56	104.62	99.57-109.67
Anterior cruciate ligament reconstruction	Female	21.08	19.4-22.76	20.15	18.81-21.49	18.76	17.53-20	22.25	20.71-23.78	12.66	11.77-13.55	15.14	14.03-16.25
	Male	32.5	30.25-34.75	30.5	28.42-32.58	27.25	25.41-29.08	26.76	24.89-28.64	15.73	14.61-16.86	17.53	16.25-18.81
	Total	26.64	24.72-28.55	25.19	23.51-26.86	22.89	21.4-24.39	24.45	22.77-26.12	14.16	13.15-15.16	16.31	15.12-17.51

ACL = anterior cruciate ligament, MR = meniscal repair, RCR = rotator cuff repair

a Rotator cuff repair.

b Meniscal repairs/meniscectomy.

c Anterior cruciate ligament reconstruction.

**Table 5:** Procedures Rate for Shoulder and Knee Arthroscopy by Age Group

Procedure	Age Group	2016		2017		2018		2019		2020		2021	
		Procedure Rate	95% Confidence Interval	Procedure Rate	95% Confidence Interval	Procedure Rate	95% Confidence Interval	Procedure Rate	95% Confidence Interval	Procedure Rate	95% Confidence Interval	Procedure Rate	95% Confidence Interval
Shoulder arthroscopy	18-35	46.03	42.77-49.29	42.46	39.68-45.24	38.42	35.95-40.88	40.56	37.87-43.26	26.86	25.13-28.58	28.23	26.42-30.03
	35-55	150.77	142.28-159.26	142.29	134.63-149.94	131.38	124.28-138.49	125.58	119.04-132.13	103.60	98.16-109.03	103.62	98.04-109.20
	55-75	249.29	235.07-263.51	242.68	230.01-255.34	233.1	221.04-245.16	228.14	216.75-239.52	192.85	183.24-202.46	193.80	183.91-203.7
	Older than 75	77.54	71.49-83.59	71.94	67.45-76.44	72.24	67.97-76.52	69.29	65.59-72.97	53.09	50.09-56.09	57.57	54.27-60.88
	Total	139.64	131.7-147.58	133.92	126.91-140.93	126.84	120.27-133.41	124.34	118.05-130.64	101.84	96.53-107.16	103.71	98.18-109.24
Rotator cuff repair	18-35	4.1	122.59-138.06	3.9	114.18-128.57	3.21	103.96-117.15	2.49	2.31-2.68	2.29	2.12-2.45	2.31	2.21-2.49
	35-55	63.38	236.81-260.89	60.7	214.19-235.8	56.64	193.35-213.17	46.79	44.13-4949.45	42.46	40.05-44.88	42.13	39.75-44.51
	55-75	152.19	275.79-304	150.81	248.24-272.56	146.05	224.54-246.69	122.72	115.68-129.77	105.96	100-111.92	107.08	100.9-113.18
	Older than 75	50.19	81.16-91.84	47.37	74.49-83.63	47.68	69.5-77.35	38.71	36.24-41.18	30.68	28.63-32.72	32.74	30.62-34.86
	Total	68.34	200.6-220.79	67.23	182.39-200.3	64.79	165.29-181.66	54.33	51.27-57.39	47.62	44.89-50.34	48.47	45.67-51.26
Knee arthroscopy	18-35	130.32	3.39-4.81	121.38	3.42-4.38	110.55	2.95-3.48	115.92	108.82-123.03	91.89	86.57-97.21	104.38	98.25-110.52
	35-55	248.85	59.69-67.06	224.99	57.28-64.12	203.26	53.42-59.87	208.9	198.8-219	175.07	166.83-183.31	177.82	169.02-186.63
	55-75	289.9	143.31-161.08	260.4	142.47-159.15	235.61	137.9-154.2	236.73	226.16-247.3	189.75	181.14-198.35	189.17	180.13-198.21
	Older than 75	86.5	46.41-53.97	79.06	44.2-50.54	73.43	44.54-50.83	72.94	69.42-76.47	51.81	49.14-54.49	55.46	52.49-58.42
	Total	210.7	64.33-72.34	191.34	63.53-70.93	173.48	61.23-68.34	177.29	168.99-180.63	143.73	136.74-150.72	149.17	141.57-156.76
Meniscal repair/meniscectomy	18-35	76.27	71.71-80.82	71.48	67.25-75.72	65.29	61.31-69.26	52.55	49.2-55.9	41.58	39.19-43.97	47.59	44.76-50.43
	35-55	196.52	187.11-205.94	176.53	168.07-184.98	161.05	153.11-168.99	144.9	137.97-151.83	122.75	117.12-128.39	123.33	117.30-129.36
	55-75	261.54	249.16-273.91	234.41	223.29-245.53	212.77	202.55-222.98	197.5	188.53-206.48	158.59	151.28-165.91	158.04	150.35-165.73
	Older than 75	78.05	73.74-82.36	71.47	67.34-75.61	66.25	62.61-69.9	61.24	58.13-64.35	44.03	41.65-46.41	46.81	44.18-49.44
	Total	167.9	160.04-175.76	152	144.9-159.09	138.69	132.09-145.28	124.95	119.19-130.71	101.92	96.94-106.78	104.62	99.34-109.90
Anterior cruciate ligament reconstruction	18-35	51.47	48.04-54.9	49.66	46.34-52.99	45.3	42.23-48.36	36.03	33.4-38.65	27.61	25.68-29.53	32.98	30.64-35.32
	35-55	27.43	25.52-29.34	26.09	24.29-27.89	23.92	22.31-25.53	19.45	17.99-20.9	15.72	14.60-16.84	17.47	16.17-18.78
	55-75	5.39	3.73-7.05	4.53	4-5.06	4.23	3.86-4.6	3.04	2.71-3.38	2.56	2.29-2.82	2.71	2.39-3.03
	Older than 75	1.01	−0.33-2.34	0.3	0.04-0.57	0.17	0.01-0.33	0.28	0.19-0.36	0.05	0.08-0.01	0.05	0.01-0.09
	Total	26.64	24.72-28.55	25.19	23.51-26.86	22.89	21.4-24.39	18.15	16.83-19.47	14.16	13.13-15.18	16.31	15.09-17.53

### Demographic Characteristics

#### Sex

**Figure 1:**
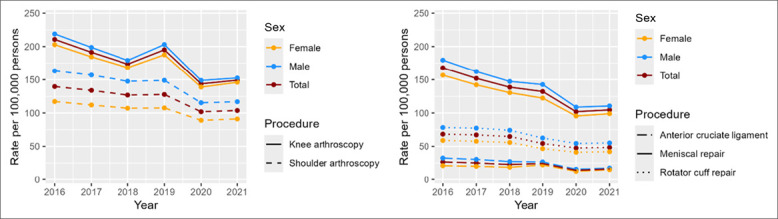
Graph showing trends in procedure rates by sex (2016 to 2021).

Both the procedure estimates and rates in the male group were higher than those in the female group for shoulder arthroscopy, RCR, MR, and ACLR across years (Tables 2 and 4 and Figure 1). However, for knee arthroscopy (except for MR and ACLR), the procedure rate in the female group was markedly higher than in the male group across years (Table 4 and Figure 1). From 2016 to 2021, the rate of shoulder arthroscopy in male patients significantly decreased from 163.23 to 116.88 (per 100,000 persons). However, there was an upsurge to 149.01 (per 100,000 persons) in 2019 compared with the previous years. Similarly, the rate of knee arthroscopy in male patients significantly decreased from 218.93 to 152.46 (per 100,000 persons) from 2016 to 2021, although rates increased in 2019 to 202.97 (per 100,000 persons; Table 4 and Figure 1). Correspondingly, procedure rates of shoulder (except RCR), RCR, knee arthroscopy (except MR and ACLR), MR, and ACLR decreased across sexes from 2016 to 2021 (Table 4 and Figure 1).

#### Age

**Figure 2:**
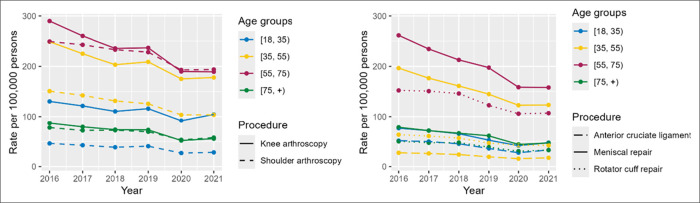
Graph showing trends in procedure rates by age group (2016 to 2021).

Both the procedure estimates and rates in the 55 to 75 age group were the highest followed by 35 to 55 and older than 75 age groups for shoulder arthroscopy, RCR, knee arthroscopy, and MR from 2016 to 2021 (Tables 3 and 5 and Figure 2). However, the procedure rate for ACLR was the highest in the 18 to 35 age group across 2016 to 2021. From 2016 to 2021, the rate of shoulder arthroscopy in the 55 to 75 age group significantly decreased from 249.29 to 193.80 (per 100,000 persons); however, there was an upsurge to 228.14 (per 100,000 persons) in 2019 compared with the previous years. Similarly, the rate of knee arthroscopy significantly decreased from 289.9 to 189.17 (per 100,000 persons) from 2016 to 2021; however, the year 2019 reported an increase in the rate to 236.73 (per 100,000 persons; Table 5 and Figure 2). In parallel, procedure rates of RCR, MR, and ACLR demonstrated similar trends (Table 5 and Figure 2).

#### Time Trends

**Figure 3:**
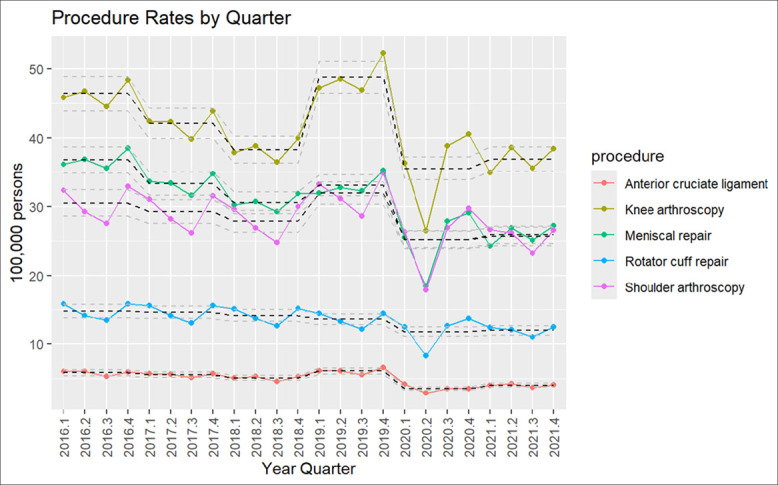
Graph showing knee and shoulder arthroscopy procedure rates by quarter.

The lowest procedure rate occurred in the third quarter and the highest occurred in the fourth quarter, a pattern observed across the years, except in 2020. In 2016, procedure rates decreased to 44.54 (per 100,000 persons) in the third quarter and increased to 48.35 (per 100,000 persons) in the fourth quarter for knee arthroscopy and decreased to 27.55 (per 100,000 persons) in the third quarter and increased to 32.89 (per 100,000 persons) in the fourth quarter for shoulder arthroscopy. In 2021, procedure rates decreased to 36.00 (per 100,000 persons) in the third quarter and increased to 38.93 (per 100,000 persons) in the fourth quarter for knee arthroscopy. Similarly, rates decreased to 23.50 (per 100,000 persons) in the third quarter and increased to 26.86 (per 100,000 persons) in the fourth quarter for shoulder arthroscopy in 2021 (Figure 3).

**Figure 4:**
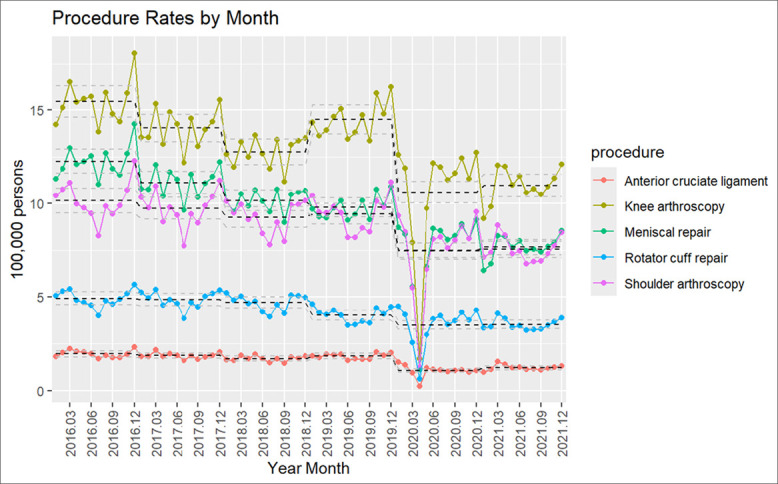
Graph showing knee and shoulder arthroscopy procedure rates by month.

Over the years, knee and shoulder arthroscopy demonstrated a similar trend in monthly rates, with July having the lowest and December having the highest number of procedure rates, with the exception of 2020 (which had the lowest rates in April-May and the highest rate in December). In 2016, knee arthroscopy showed the highest rate (18.07 per 100,000 persons) in December and the lowest (13.83 per 100,000 persons) in July. Similarly, in 2021, knee arthroscopy showed the highest rate (12.25 per 100,000 persons) in December and the lowest (11.61 per 100,000 persons) in July (Figure 4).

Overall, after comparing trends across the years, procedure rates significantly decreased for MR; knee and shoulder arthroscopy with a *p*-value of 0.0001. Trends for RCR and ACLR did not show notable change when comparing 2016 and 2021. The second quarter (month 3 to 6) of 2020 showed the lowest rates for all the procedures. The highest drop in the procedure rate was observed for the knee arthroscopy, shoulder arthroscopy, and MR, followed by RCR (Figures 1 and 2).

## Discussion

We analyzed the Nationwide Ambulatory Surgery Sample (NASS) dataset from 2016 to 2021 to estimate the procedure rates and time trends of knee arthroscopy and shoulder arthroscopy done on an ambulatory basis. Based on our analysis, we observed an overall declining trend in ambulatory surgery rates. Although 2019 did show a modest increase in the rates, the overall trend supports the hypothesis of declining ambulatory surgery rates in the years leading up to and including the pandemic period. There was a double U-shaped decline in the estimates of shoulder arthroscopy (except RCR), MR, and knee arthroscopy, with an initial decline from 2016 to 2018 followed by a peak in 2019 and then sudden decline in 2020 to the lowest rate for each procedure followed by a subsequent rise. There were markedly more RCR, MR, and ACLR done among male patients as compared with female patients. However, for knee arthroscopy (except for MR and ACLR), the procedure rate in female patients was markedly higher than that in male patients. The procedure rate of shoulder arthroscopy and knee arthroscopy, RCR, and MR/meniscectomy were the highest among 55 to 75-year-old patients. However, the procedure rate for ACLR was the highest in the 18 to 35-year age group across all years.

Over the years, previous state data have shown markedly lower knee arthroscopy rates; one study reported a decrease in the rate of knee arthroscopy in the state of Florida from 449 procedures per 100,000 people in 2002 to 345 per 100,000 people in 2015 for patients with osteoarthritis, meniscal tears, and knee pain in the state of Florida.^16^ Notable downward trends in knee arthroscopy rates were also reported in California, Iowa, Maryland, New Jersey, Michigan, and Nebraska from 2006 to 2016.^16,17^ By contrast, RCR rates have continued to rise in several states over time. This divergence may be attributed to the availability of high-level evidence from large randomized trials questioning the efficacy of knee arthroscopy, whereas comparable randomized evidence evaluating the effectiveness of RCR remains limited.^17^ Similarly, Amin et al and Potts et al reported a decreasing rate for knee arthroscopy using state data from 1998 to 2006 and American Board of Orthopaedic Surgery data from 1999 to 2009, respectively.^18-20^ Thus, in terms of trends in knee arthroscopy procedures, our finding aligns with the previous literature.

It is interesting that the surgeries peaked in 2019 before dropping in 2020. In 2020, Moree et al evaluated the Medicare reimbursement patterns for popular arthroscopic procedures from 2000 to 2019, and the results showed a notable decline in inflation-adjusted payment rates. In particular, the study discovered that throughout this time, Medicare reimbursement for the 20 most common arthroscopic operations fell by over 30%, increasing operating room access which would increase the number of cases.^21^ In conclusion, the 2019 modifications affected both knee and shoulder surgeries and were a part of a larger pattern of decreasing Medicare compensation for arthroscopic procedures, potentially increasing operating room access and number of cases.

After 2019, there was a sharp decrease in the estimates and rates for the shoulder arthroscopy, RCR, MR, knee arthroscopy, and ACLR procedures in 2020 which is largely related to the COVID-19 pandemic. The Centers for Medicare and Medicaid Services, the American Society of Anesthesiologists, the Center for Disease Control and Prevention (Centers for Disease Control and Prevention), and the Society for Ambulatory Anesthesia published a series of guidelines based on the evidence, recommending that all elective and nonessential procedures can be delayed, limiting exposure to the virus^22-25^ which correlates with our findings of the lowest estimates and rates of all procedures for the March-May 2020 time frame. In 2022, Mafi et al^26^ reported trends in US ambulatory care patterns during the COVID-19 pandemic and concluded that there was a significant decrease in ambulatory care service between March and April 2020, followed by an increase in procedure rates afterward.

Furthermore, in 2022, Khan et al^27^ reported a decrease in shoulder arthroplasty volume per 1000 Medicare beneficiaries by 14% during the COVID-19 pandemic using Centers for Medicare and Medicaid Services inpatient and outpatient claims database from 2019 to 2020, indicating an overall decreasing surgical trend. Liu et al reported a reduction in number of ACLR and rotator cuff surgeries performed in a local hospital, indicating a decreasing trend of sports and arthroscopic surgeries.^28^ However, our trends for ACLR showed less variation across 2016 to 2021 with an insignificant decline in rates in 2020, which can be explained by the acute nature of ACL injuries necessitating more urgent surgical intervention. Furthermore, for both the shoulder and knee arthroscopy, the procedure rates were at the highest in December and lowest in July. This is likely because of health insurance policies, as deductibles usually reset at the end of the year^29^ and hence many patients may seek elective healthcare services before reset at the beginning of the new year. In addition, pursuing elective orthopaedic surgery in December, when patients and caregivers tend to have vacation time, makes it easier for patients to undergo and recover from procedures because of the time off around the holidays.^30^

Degen et al^31^ evaluated a longitudinal trend in knee arthroscopy using the American College of Surgeons National Surgical Quality Improvement Program database from 2006 to 2016, and reported an increase in the knee arthroscopy utilization rate with increasing average age. Similarly, Strope et al^32^ evaluated the 2005 State Ambulatory Surgery Database for Florida in a cross-sectional study and reported a markedly higher procedure rate of ambulatory surgeries (orthopaedic, urologic, ophthalmologic, and gastrointestinal procedures) in the age group of 60 to 79 years compared with other age groups. Our study found a similarly higher rate in shoulder arthroscopy and knee arthroscopy procedures in the age group of 55 to 75 years. Mazzocca et al^33^ reported a higher number of knee and shoulder arthroscopy procedure rates in female patients (59.3%) compared with male patients (40.7%)_._ However, our study reported higher rates of shoulder arthroscopy, RCR, knee arthroscopy, MR/meniscectomy, and ACL reconstruction procedures in male patients as compared with female patients. Thus, in terms of sex, results of our analysis do not align with the findings from previously reported studies. Our study has various strengths. It uses the HCUP-NASS database, which is designed to represent approximately 2,881 hospital-owned facilities across 34 states and the District of Columbia.^34^ The database used for this analysis was weighted to generate national and regional estimates.

## Limitations

There are several limitations to this study. The NASS dataset includes all ambulatory procedures from hospital-owned facilities. Although most knee and shoulder arthroscopies done in the United States occur in the ambulatory setting, any shoulder or knee arthroscopic procedures done as inpatient cases would not be captured in this dataset which could contribute to the lower number of procedures observed in our study. Similarly, there could be selection bias, as the dataset does not represent the entire cohort of ambulatory surgery patients, since it is limited to hospital-owned facilities. Since the data provided in the NASS file are at the encounter level, patient-level data are lacking, limiting some patient demographics and specificity regarding specific procedures because of the absence of HCPCS level II codes, such as those for ambulance services, durable medical equipment, and prosthetic codes. The database is dependent on accurate coding for inclusion in the dataset and any errors could lead to result inaccuracies. The NASS dataset is updated periodically which can result in a lag in the availability of the most recent data. Lastly, there are no other studies that have used the HCUP-NASS database previously; therefore, it is difficult to make direct comparisons with other studies.

## Conclusion

There was a nonlinear decrease in the estimates of ambulatory shoulder arthroscopy, RCR, MR/meniscectomy, knee arthroscopy, and ACL reconstruction procedures from 2016 to 2021 with peaks in 2016 and 2019 and a low point in 2020.
